# Complete Genome of *Acinetobacter baumannii* N4-Like Podophage Presley

**DOI:** 10.1128/genomeA.00852-13

**Published:** 2013-12-05

**Authors:** Nicholas G. Farmer, Thammajun L. Wood, Karthik R. Chamakura, Gabriel F. Kuty Everett

**Affiliations:** Center for Phage Technology, Texas A&M University, College Station, Texas, USAa; Department of Biochemistry and Molecular Biology, Pennsylvania State University, University Park, Pennsylvania, USAb

## Abstract

*Acinetobacter baumannii* is an emerging multidrug-resistant nosocomial pathogen. Bacteriophages may be useful as an alternative method of treatment against this and other multidrug-resistant bacteria. Here, we present the complete genome sequence of *A. baumannii* phage Presley, an N4-like podophage.

## GENOME ANNOUNCEMENT

*Acinetobacter baumannii* is a Gram-negative opportunistic pathogen. It earned the nickname “Iraqibacter” due to its emergence in military medical facilities in the Middle East (1). Currently, *A. baumannii* infections are a problem in civilian hospitals due to the bacterium's desiccation resistance and its ability to develop resistance to many antibiotics (2). Bacteriophages, such as the N4-like podophage Presley, described here, may be an alternative to the use of antibiotics in the fight against multidrug-resistant pathogens like *A. baumannii*.

Bacteriophage Presley was isolated from a sewage sample collected in College Station, TX. Phage DNA was sequenced using 454 pyrosequencing at the Emory GRA Genome Center (Emory University, Atlanta, GA). The trimmed FLX Titanium reads were assembled to a single contig at 293-fold coverage using the Newbler assembler version 2.5.3 (454 Life Sciences) with the default settings. The contig was confirmed to be complete by PCR. Genes were predicted using GeneMarkS (3) and corrected using software tools available on the Center for Phage Technology (CPT) portal (https://cpt.tamu.edu/cpt-software/portal/). Morphology was determined using transmission electron microscopy performed at the Texas A&M University Microscopy and Imaging Center.

Presley has a unit genome of 77,181 bp, with 94 unique coding sequences (CDSs), an overall G+C content of 37.8%, and a coding density of 95.8%. Of the 94 CDSs, more than half (54 genes) are hypothetical novel genes, while 23 are homologous to genes of *Escherichia coli* phage N4 (accession no. NC_008720). Processing of the raw sequencing reads using the Pause method (https://cpt.tamu.edu/cpt-software/releases/pause/) revealed a terminal repeat of 611 bp, which is slightly smaller than the repeats found in N4-like *Pseudomonas* phages LUZ7 (660 bp; accession no. NC_013691) and LIT1 (655 bp; accession no. NC_013692) (4).

The genome of phage Presley encodes proteins corresponding to replication, transcription, DNA packaging, and morphogenesis functions. For genome injection, Presley encodes two N4-like RNA polymerases (a virion-associated RNA polymerase [vRNAP] that is injected with the first part of the genome and RNA polymerase II [RNAP II] found among the early genes) and a single-stranded DNA-binding protein (SSB) (5, 6). In phage N4, the vRNAP translocates a fraction of the phage genome into the host cell via the transcription of early genes, which includes RNAP II. The newly synthesized RNAP II is recruited to the DNA by SSB and translocates the remainder of the genome. Since Presley encodes these three proteins, it presumably accomplishes DNA injection by the same mechanism. The N4-like DNA replication proteins present include DNA helicase, DNA polymerase, DNA primase, Holliday junction resolvase, and two ATPases. Presley also encodes an N4-like major capsid protein, N4 gp52 and gp54-like structural proteins, portal protein, and large terminase.

Outside of its homology with N4, Presley encodes a tail fiber with a transglycosylase domain and major tail protein that was identified by an Ig domain (7). The lysis cassette consists of an endolysin (*N*-acetylmuramoyl-l-alanine amidase), inner and outer membrane spanin components, and a holin with one transmembrane domain in an N-out C-in topology.

### Nucleotide sequence accession number.

The genome sequence of phage Presley was contributed as accession no. KF669658 to GenBank.
